# Gender-Dependent Effects of Maternal Immune Activation on the Behavior of Mouse Offspring

**DOI:** 10.1371/journal.pone.0104433

**Published:** 2014-08-11

**Authors:** Ingrid C. Y. Xuan, David R. Hampson

**Affiliations:** 1 Department of Pharmaceutical Sciences, Leslie Dan Faculty of Pharmacy, University of Toronto, Toronto, Canada; 2 Department of Pharmacology and Toxicology, Faculty of Medicine, University of Toronto, Toronto, Canada; Western University of Health Sciences, United States of America

## Abstract

Autism spectrum disorders are neurodevelopmental disorders characterized by two core symptoms; impaired social interactions and communication, and ritualistic or repetitive behaviors. Both epidemiological and biochemical evidence suggests that a subpopulation of autistics may be linked to immune perturbations that occurred during fetal development. These findings have given rise to an animal model, called the “maternal immune activation” model, whereby the offspring from female rodents who were subjected to an immune stimulus during early or mid-pregnancy are studied. Here, C57BL/6 mouse dams were treated mid-gestation with saline, lipopolysaccharide (LPS) to mimic a bacterial infection, or polyinosinic:polycytidylic acid (Poly IC) to mimic a viral infection. Autism-associated behaviors were examined in the adult offspring of the treated dams. Behavioral tests were conducted to assess motor activity, exploration in a novel environment, sociability, and repetitive behaviors, and data analyses were carried independently on male and female mice. We observed a main treatment effect whereby male offspring from Poly IC-treated dams showed reduced motor activity. In the marble burying test of repetitive behavior, male offspring but not female offspring from both LPS and Poly IC-treated mothers showed increased marble burying. Our findings indicate that offspring from mothers subjected to immune stimulation during gestation show a gender-specific increase in stereotyped repetitive behavior.

## Introduction

Autism spectrum disorders (ASDs) are a behaviorally defined class of neurodevelopmental disorders characterized by two core symptoms: 1) social and communication impairments, and 2) ritualistic repetitive behaviors [1]. The auxillary symptoms associated with ASDs are highly heterogeneous and vary in severity. Examples include hyperactivity, motor impairments, intellectual disability, and anxiety [2], [3], [4]. The diagnosis of ASD shows a marked male preponderance with incidence estimates of male-to-females ranging from 3-fold to 11-fold [5], [6], [7]. As noted by Kim et al., 2013, the male prevalence of ASD is similar to other early onset anti-social disorders such as developmental language disorder and attention-deficit hyperactivity disorder [8], [9]. The reasons for this high male prevalence remain unclear.

About 10–20% of ASD cases can be attributed to genetic factors, while the causes of the other 80–90% are unknown [10]. Since ASD is typically diagnosed at 1–3 years of age, the prenatal environment may be particularly important for the pathogenesis of this disorder. Hallmayer et al., 2011 compared the incidence of ASD between monozygotic and dizygotic twins and found that approximately 58% of the liability to autism could be explained by environmental factors common to twins [11]. Environmental factors such as infection may trigger the maternal and/or fetal immune systems and alter the normal brain maturation process, resulting in brain pathologies and behavioral abnormalities. Epidemiological studies suggest that maternal immune activation (MIA) during pregnancy may be a risk factor for ASD. Based on a large Danish cohort (N = 1.6 million), Atladóttir et al., 2010 found that maternal viral and bacterial infection (specifically respiratory infection) during the first trimester increased the risk of ASD in the offspring by two and three-fold, respectively [12]. The same group also reported that a febrile episode lasting more than seven days increased the risk of infantile autism by three-fold in the first trimester and four-fold in the second trimester [13]. In a separate study based on a large Finnish cohort (N = 1.2 million), an increased level of maternal C-reactive protein, a biomarker for inflammation, was shown to be significantly associated with ASD diagnosis in the offspring [14].

The involvement of immune activation in ASD is further supported by reported increased serum cytokine levels in ASD patients [15], and by elevated expression of cellular markers for microglia and astrocytes in post-mortem brain tissue from persons with ASD [16], [17], [18], [19]. Rodent-based MIA models of neurodevelopmental disorders were developed to mimic aspects of these phenomena in a laboratory setting. It is important to note however that in addition to ASD, the MIA paradigm has also been used to model schizophrenia, which has similarly been associated with prenatal immune disturbances in the patient population [20], [21], [22]. Maternal immune activation is typically induced by injecting lipopolysaccharide (LPS) or polyinosinic:polycytidylic acid (Poly IC) into pregnant female rats or mice during gestation to mimic a bacterial or viral infection, respectively [23]. ASD-like behaviors most often studied include measuring impairments in social interactions, increased repetitive behavior assessed using the marble burying test or quantitating self-grooming events, and the examination of anxiety levels in the open field test [24], [25], [26], [27].

The objective of the present study was to compare the effects of MIA on the behavior of male vs. female mouse offspring. We examined the effects of MIA induced by either LPS, or by Poly IC. Our findings indicate that both the gender of the mouse, and the type of immune stimulus influence a subset of autistic behaviors of MIA offspring.

## Experimental Procedures

### Induction of MIA

All animal experiments were carried out in accordance with the guidelines set out by the Canadian Council on Animal Care and were approved by the University of Toronto Animal Care Committee. All experiments were carried out using C57BL/6 mice housed in cages containing 4 mice and with lights on at 6:00 a.m. and off at 8:00 p.m. The mice were mated overnight and checked for the presence of a vaginal plug at 9 a.m. each day; the day that the vaginal plug was observed was marked as embryonic day 0.5 (E0.5). A total of 5 litters were used from Poly IC-injected dams, 6 litters from LPS-injected dams, and 6 litters from saline-injected dams.

The LPS injection protocol used was a modification of the multiple injection procedure used in rats by Girard et al., 2010. For LPS treated mouse dams, two intra-peritoneal (i.p.) injections of 75 µg/kg LPS (5 µl/g lipopolysaccharide O127:B8; Sigma Cat. # L3129) were given on both days E11.5 and E12. For Poly IC, dams were injected i.p. with 20 mg/kg polyinosinic:polycytidylic acid (Poly IC) potassium salt (5 µl/g; Sigma Cat. #P9582) once on E12.5. Poly IC was dissolved in saline at 4 mg/mL (based on the weight of Poly IC itself, which was 10% of total weight of the salt). Separate groups of control female mice were injected with saline on E11.5 and E12.5 (LPS control), or only on E12.5 (Poly IC control).

### Behavioral testing

All pups were housed with their mother until postnatal day (PND) 21 when they were weaned and ear tagged. After weaning, two to four pups of the same sex and treatment were housed together per cage. In cases where only a single female or male was born in one litter, it was housed with other mice of same sex and similar age (from a different treatment group). All animals were left undisturbed except for biweekly cage changes and behavior tests. The order of behavioral testing was as follows: 1) motor activity at postnatal 6–7 weeks; 2) modified 3-chamber paradigm social test at 7–8 weeks; 3) self-repetitive grooming at 7–8 weeks scored based on recorded videos during social testing; 4) marble burying test at 9–10 weeks. All behavioral testing with the exception of the social tests was conducted between 9 am and 2 p.m.; social testing was performed between 10 a.m. and 5 p.m.

### Motor Activity

Motor activity was measured using an automated VersaMax animal activity monitoring system (AccuScan Instruments, Inc., U.S.A.) as previously described [28]. The detection system consisted of a 42×42×30 cm plexiglas box placed inside the activity monitor that emitted 16×16×16 laser beams, each separated by 2.5 cm; the system measured the animal's activity by tracking the number of beams that were broken. Two smaller plexiglas boxes (21×21×21 cm) were placed at diagonal corners inside the larger plexiglas box for activity measurement of each individual mouse. All animals were habituated under dim lighting (the room was evenly lit by three 14 W incandescent light fixtures) for at least one hour prior to testing. Before each test, the smaller box was wiped thoroughly with Virox (0.4% hydrogen peroxide) followed by water. The animal was gently placed in the center of the smaller box with a lid; the test was performed under dim lighting and the data generated by the monitor was tabulated every 5 minutes for an hour. The following parameters were analyzed: total distance travelled, horizontal movement number, vertical movement number, per cent of total distance travelled in the center zone (center defined as 10.5 cm×10.5 cm), and time spent in the center zone.

### Social Interaction and Social Preference Tests

A modified three-chamber protocol was used based on that described by Ramsey et al., 2011 with some modifications [29]. The test was conducted under dim lighting (approximately 40 lux). A digital camera was mounted on the ceiling approximately one meter above the social apparatus to track the locomotion of the animal. Rather than physically separating the chambers, we virtually defined two identical circular zones (diameter: 19.8 cm) in a white plexiglas box (61.7×40.8×23 cm) using Viewer^2^ software (BIOBSERVE GmbH, NJ). These two zones were side-by-side to each other and equidistant from the edge of the box (7.1 cm lengthwise, 10.5 cm widthwise). An empty white wire cage (top diameter 7.5 cm, bottom diameter 10 cm, height 10 cm) was placed at the center of each zone, covered by a white circular disk (diameter: 10 cm) and held down by a standard 400 ml glass beaker. The beaker prevented the test animals from climbing on top of the wire cages. Two removable, white cardboard dividers (40×23 cm) were inserted into the middle of the box to shield both zones. The activity of the animal in each zone was recorded every 2 minutes for 10 minutes.

The stimulus animals (untreated wild-type C57BL/6 mice age and sex matched with the test animal) were kept at the opposite corner from the test animal to ensure its novelty. Stimulus animals were habituated for 30–60 minutes in their respective home cage under dim lighting as previously described. Each animal was then allowed to habituate to the inside of a clean cylindrical wire cage placed inside the white plexiglas box. A maximum of six stimulus animals were habituated at the same time for two 15-minute sessions. The stimulus animals were used for subsequent social testing if no persistent aggressive behavior was observed (e.g. climbing and/or biting of the cage).

Test animals were habituated for at least one hour in their home cages under dim lighting as previously described. Before each test, all apparatus were wiped vigorously with Virox, followed by water. To begin, the test animal was gently placed between the dividers. Then the dividers were simultaneously lifted, exposing the animal to the two zones, each with an empty wire cage held down by a beaker as previously described. After the animal was allowed to habituate for 10 minutes, it was then isolated between the two dividers again, shielding it from the two zones. If side preference was present (i.e. the difference in time spent between the two zones was more than 30 seconds), the animal was returned to home cage and tested the next day. Otherwise, the social interaction test was performed. One stimulus animal (of same sex and similar age) was placed in one of the wire cages while a novel object was placed in the other cage. The designation of the zones was determined at random. The novel object used was a green Biobag tied into a knot placed on top of a roll of green tape (6 cm diameter). The dividers were then simultaneously lifted and the animal was allowed to explore for 10 minutes. During this 10 minute period, the time spent in each zone was recorded. At the end of the social interaction test, the animal was again isolated between the dividers to shield it from both zones. Subsequently, the social preference test was performed where a novel stimulus mouse (of same sex and similar age) was placed in the non-social zone, replacing the novel object; this then became the non-familiar zone. The dividers were then lifted simultaneously and the animal was allowed to explore for 10 minutes while the time spent in each zone was recorded.

A t-test was performed to determine if there was a significant preference for one zone over the other in the social interaction and social preference tests; each treatment group was evaluated separately [30], [31].

### Marble Burying Assay

The marble burying assay, a test for repetitive/compulsive behavior, was conducted at 10 weeks of age as previously described [32]. Clean cages (29.5×17.5×12.5 cm) were filled 5 cm deep with corn cob bedding. Twenty identical navy blue marbles (1.2 cm diameter) were placed in a 4×5 rectangular matrix occupying 2/3 of the cage. The animals were first habituated in the biosafety cabinet class A/B for at least 30 minutes with the blower on as background noise. After habituation, the test mouse was gently placed into 1/3 of the cage without the marbles and a lid was immediately placed over the cage. Individual test animals were allowed to explore the cage for a 30 minute test period after which they were returned to the home cages. A marble was considered buried when more than 50% of its surface area was covered by the bedding. The marbles were cleaned with Virox and rinsed with water between each test.

### Grooming analysis

Self-grooming behavior entails licking and scratching of any body parts of the mouse [33]. The total time spent grooming (in seconds) was recorded manually based on previously recorded video during the first 5 minutes of habituation in the three-chamber paradigm.

### Statistical Analyses

Statistical analyses were performed using SPSS version 18. For thigmotaxis, marble burying, and grooming, a two-way ANOVA analysis was used to determine differences among multiple groups with two variables (i.e. gender and treatment), followed where appropriate by the Bonferroni post hoc test. For motor activity analysis, a three way ANOVA was carried out using treatment x gender x time interval. A two-tailed Student's t-test using GraphPad Prism was used for within group comparisons (e.g. saline social zone vs. saline non-social zone) in the social interaction and preference tests. Grubbs' test was used to eliminate outliers. For all statistical tests, *p*<0.05 was considered significant and 0.05<*p*<0.1 was considered as a trend towards a significant effect.

## Results

**Figure 1:**
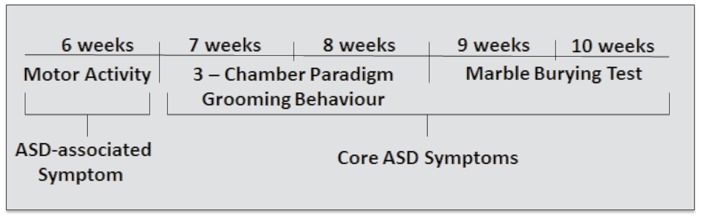
Timeline for behavioral testing. The age of the mice in weeks is indicated.

To avoid potential complications from analyzing mice during the period when the brain is rapidly maturing, behavioral testing began in late adolescent mice at 6 weeks of age (Fig. 1). This corresponds to an age just prior to brain maturation in the mouse at 7–8 weeks postnatal. To reduce the effects of repeated measures, behavioral analyses were conducted one week apart. Video recording of grooming was conducted during the habituation period of social testing as well as during the social preference test.

### The effect of MIA on body weight

The body weights of MIA offspring were measured on PND 46 as an indicator of their overall health. Male saline, LPS, and Poly IC mice weighed 21.8±0.5, 23.0±0.2, 21.7±0.5 grams respectively; female saline, LPS, and Poly IC mice weighed 18.0±0.3, 17.6±0.4, and 17.8±0.6 grams (mean ± S.E.M; n = 7–17 per group). As expected, male mice were heavier than female mice (*F* (1, 68) = 160.9, *p*<0.0001). However, body weight was not affected by prenatal LPS or Poly IC infection (*F* (2, 68) = 1.14, *p* = 0.3251), and there was no significant interaction between treatment and gender (*F* (2, 68) = 2.45, *p* = 0.0943).

### The effect of MIA on motor and open field activity

Locomotor and exploratory behaviors of MIA offspring were investigated by measuring horizontal activity, vertical activity, and thigmotaxis behavior in an automated activity box. All parameters were separately analyzed over three time intervals: 0 to 20, 20 to 40 and 40 to 60 minutes with the exception of thigmotaxis behaviour, which was analyzed only during the first time interval at 0 to 20 minutes. A summary of all statistical analyses is shown in Table S1.

**Figure 2:**
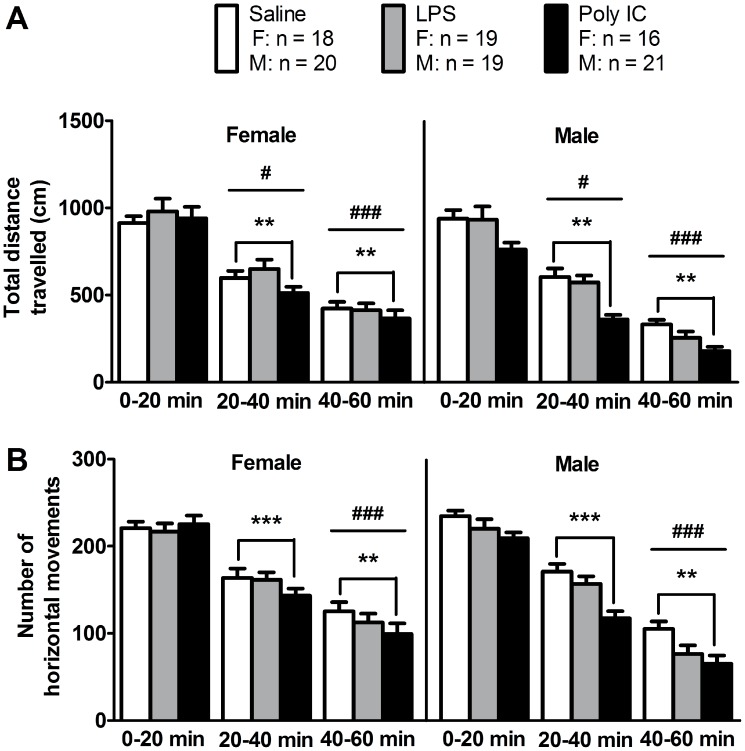
The effects of maternal immune activation on horizontal motor activity of adult offspring. Compared to saline offspring, Poly IC offspring, but not LPS offspring, showed significantly reduced (A) total distance travelled and (B) number of movements at 20–40 minutes and 40–60 minutes (**p<0.01, ***p<0.001; see Table S1 for a summary of statistical analyses). A significant gender effect was also observed at 20–40 minutes and/or 40–60 minutes (#*p*<0.05; ###*p*<0.001). Each column represents mean ± S.E.M. The number of mice tested (n) for males (m) and females (f) for each condition is indicated.

The treatment x gender interaction effect was not significant for any of the three time intervals, and prenatal LPS treatment did not affect the horizontal activity of either female or male offspring during any of the three time intervals. However, in male offspring, a significant main effect of treatment was seen where the Poly IC mice displayed reduced total distance travelled (Fig. 2A) and reduced horizontal movement number (Fig. 2B) at 20–40 minutes (total distance travelled: *F* (2, 107) = 10.81, *p*<0.001, Bonferroni post hoc test *p*<0.001; horizontal movement number: *F* (2, 107) = 9.43, *p*<0.001, Bonferroni post hoc test *p*<0.001), and 40–60 minutes (total distance travelled: *F* (2, 107) = 4.35, *p*<0.05, Bonferroni post hoc test *p*<0.01; horizontal movement number: *F* (2, 107) = 5.41, *p*<0.01, Bonferroni post hoc test *p*<0.01). The gender effect was significant for total distance travelled only during the last two time intervals (20–40 min: *F* (1, 107) = 4.72, *p*<0.05; 40–60 min: *F* (1, 107) = 24.86, *p*<0.001) and significant for movement number during the last time interval (*F* (1, 107) = 13.30, *p*<0.001).

The effect of maternal immune activation on vertical activity, measured by vertical movement number, was gender-dependent (Figure S1). There was a significant gender effect during the first two time intervals (0–20 min: (*F* (1, 107) = 20.77, *p*<0.001) and 20–40 minutes (*F* (1, 107) = 6.40, *p*<0.05)). A significant treatment effect was observed for the middle time interval only (*F* (2, 107) = 4.91, *p*<0.01). However, further post-hoc test showed that neither LPS nor Poly IC offspring significantly differed from control. The treatment x gender interaction effect for vertical movement number was not significant for any of the three time intervals.

Thigmotaxis describes the animals' innate preference to stay near the walls rather than the center of the open field. Increased thigmotaxis is indicative of increased anxiety, which is an associated symptom of ASD. To investigate this behavior in the MIA mice, we measured the activity of the mice in the center zone of the motor activity box during the first 20 minutes of the exploration period (Figure S2). There was a significant gender effect for both time spent in center (*F* (1, 104) = 5.61, *p*<0.05) and per cent of total distance travelled in the center zone (*F* (1, 104) = 9.29, *p*<0.01). The treatment main effect and treatment x gender interaction were not statistically significant.

### The effects of MIA on social behavior

**Figure 3:**
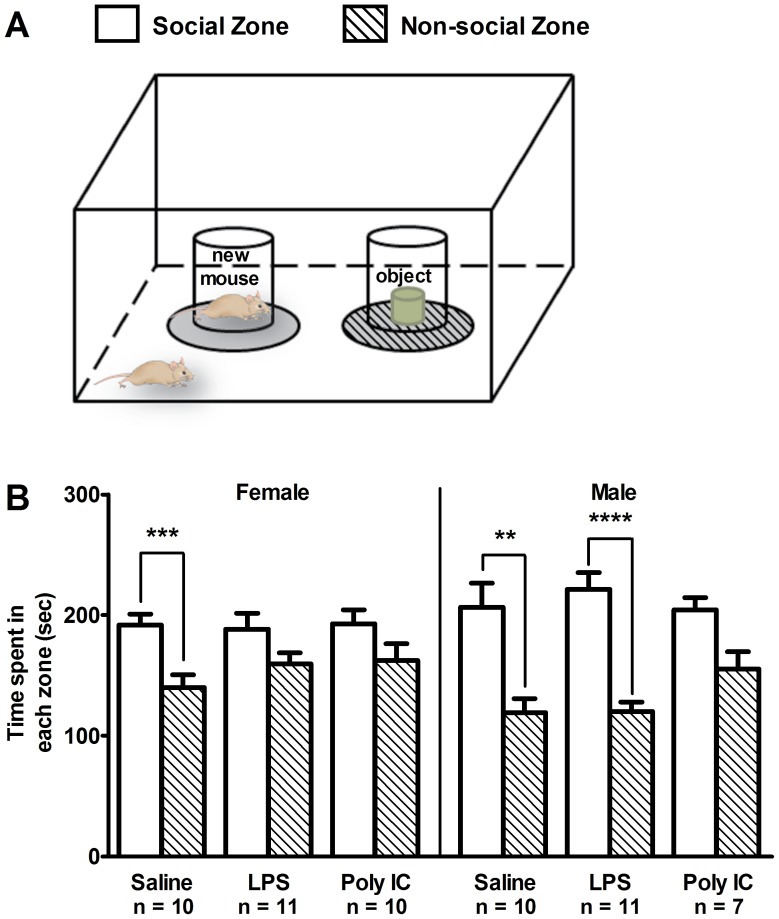
The effects of maternal immune activation on social behavior in the three-chamber social interaction test. (A) diagrammatic depiction of the experimental set-up for the social interaction test. (B) Male and female saline-treated mice showed the expected preference for the social zone (**p<0.01; ***p<0.001; ****p<0.0001). However, compared to saline-treated controls, female LPS and Poly IC offspring showed no significant preference for the social zone over the non-social zone. A loss of the normal social interaction behavior was also observed in male Poly IC mice, but not in male LPS mice compared to control mice. Each column represents mean ± S.E.M.

Social impairment is one of the core symptoms of ASD and is often linked with deficits in communication. In rodents this behavior can be assessed using three-chamber testing paradigms [34]. The test is divided into two stages. In the social interaction test, sociability was measured by comparing the time the animal spent in the zone with a novel mouse (the social zone) versus a novel object (non-social zone; Fig. 3A). In the second stage, termed the social preference test, sociability was measured by comparing the time the animal spent in the zone with a familiar mouse (the familiar zone) versus a new non-familiar mouse (the non-familiar zone, Figure S3A). In both tests, normal social behavior entailed significant preference for the social and non-familiar zones over the non-social and familiar zones, respectively.

For the social interaction test, saline-treated offspring demonstrated normal social behavior by spending significantly more time in the social zone rather than the non-social zone (Fig. 3B; female: *t* (9) = 5.09, *p*<0.001; male: *t* (9) = 3.96, *p*<0.01). Prenatal LPS caused abnormal social behavior in female offspring where the mice did not show significant preference for the social chamber (LPS: *t* (10) = 1.79, *p* = 0.1041). In contrast, male LPS offspring demonstrated normal social behaviour (LPS: *t* (10) = 9.59, *p*<0.0001). Male mice from Poly IC injected mothers showed a strong trend towards normal behavior (Fig. 3B; *t* (6) = 2.38, *p* = 0.0548).

In the social preference test, neither female (saline: *t* (9) = 1.31, *p* = 0.2222, LPS: (*t* (10) = 1.14, *p* = 0.2803, Poly IC: (*t* (9) = 1.30, *p* = 0.2261) nor male offspring (saline: *t* (9) = 1.42, *p* = 0.1893, LPS: (*t* (10) = 1.59, *p* = 0.1422), Poly IC: (*t* (6) = 1.49, *p* = 0.1870) showed a significant preference for the non-familiar zone over the familiar zone (Figure S3B). A two-way ANOVA comparison of the time spent in the non-familiar zone between different treatment groups showed no significant treatment effect, gender effect, or interaction effect.

### The effect of MIA on repetitive behaviors

**Figure 4:**
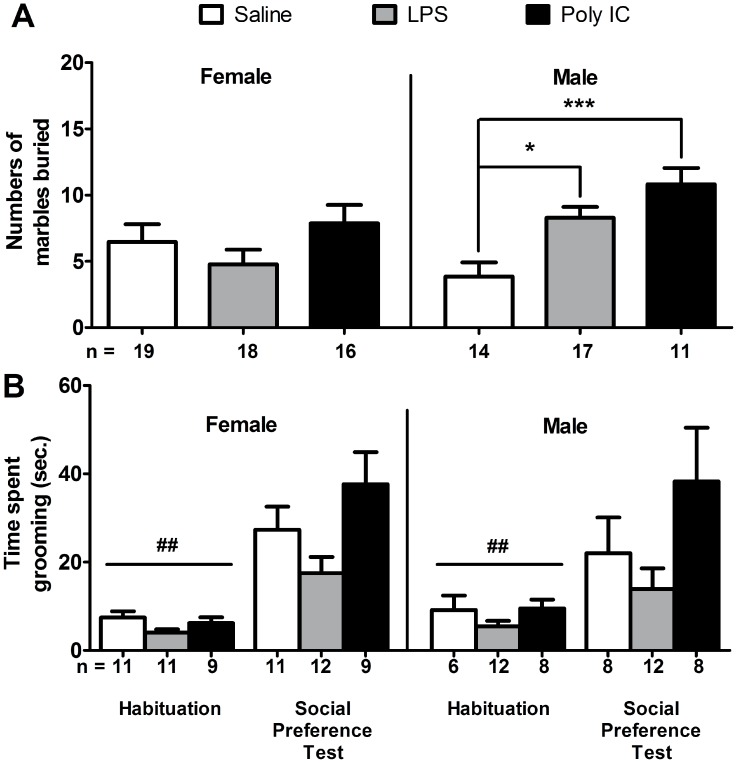
Analysis of repetitive behaviors. (A) In the marble burying test, both male LPS and male Poly IC offspring showed significantly increased repetitive behavior, whereas female LPS and female Poly IC offspring did not (*p<0.05, ***p<0.001, between treatments). (B) Self-grooming during the habituation period prior to social testing, and during the social preference test. During habituation, a significant gender main effect was observed (##p<0.01, between gender). During the social preference test, male Poly IC mice, and to a lesser extent female Poly IC mice, showed a non-significant elevation of grooming relative to saline-treated controls. Each column represents mean ± S.E.M.

Ritualistic and repetitive behavior is another core symptom of ASD. Such behavior can be assessed in rodents using the marble burying test where increased repetitive behavior is represented by more buried marbles (32). The results of the two-way ANOVA revealed a significant treatment effect (Fig. 4A; *F* (2, 89) = 5.72, *p*<0.01) where both male Poly IC (Bonferroni post-hoc test *p*<0.001) and male LPS offspring (Bonferroni post-hoc test *p*<0.05) buried significantly more marbles than compare to saline treated control male mice. The interaction between treatment x gender was significant (*F* (2, 89) = 4.09, *p*<0.05), and notably, neither LPS nor Poly IC female offspring displayed increased marble burying compared to controls (Fig. 4A).

Repetitive behavior was also evaluated by scoring the time the animal spent grooming during the first five minutes of the habituation period (i.e. exploration in open field box with two empty wire cages with no stimulus mice present) and the first five minutes of the social preference test (i.e. exploration in open field box with two wire cages, each with a stimulus mouse trapped inside) (Fig. 4B). There was a significant treatment effect for both grooming during habituation period (*F* (2, 51) = 3.76, *p*<0.05) and social preference test (*F* (2, 56) = 3.50, *p*<0.05). However, post-hoc analysis showed no statistical significance between saline and LPS or saline and Poly IC for both sexes. A significant gender effect was found only during the habituation period (*F* (1, 51) = 10.24, *p*<0.01). The treatment x gender interaction was not significant for the habituation period (*F* (1, 51) = 0.8205, *p* = 0.4459) or during social preference test (*F* (1, 56) = 0.2280, *p* = 0.7968).

## Discussion

This study was designed to investigate gender differences in mice in response to immune activators administered to the mother during pregnancy. Whereas most (but not all, see below) previous studies on MIA have either examined only male rodents, or have combined male and females together, here we directly compared the behavioral effects of LPS and Poly IC in separate groups of male and female mice. Our findings indicate that male and female offspring from mothers injected with LPS or Poly IC during mid-gestation display disparate behaviors on tests of motor activity, social interactions, and repetitive or stereotyped behaviors.

Impairments in social interactions and communication are core symptoms of ASD. Our findings revealed modest social impairments in female LPS offspring. Kirsten et al. 2010; 2012 investigated sociability in rats prenatally treated with LPS by subjecting the offspring to a novel free moving stimulus animal in a cage, and manually scoring their play behavior including sniffing, following, crawling under/over, and mounting on the stimulus rat; they found male-specific deficits in the latter two parameters only [25], [26]. These two parameters measure more aggressive behaviors that are usually more common in male rodents. Our test conditions were less aggressive because the stimulus mice were constrained in wire cups; this could have contributed to the differences that we observed between male and female animals. Importantly, Kirsten et al., 2010 were only able to detect deficits in social interaction when they isolated the rats (i.e. one rat per cage) for a week prior to testing in order to increase the rats' social motivation (not done in the present study) [25]. Therefore, due to the differences in experimental conditions, the lack of social interaction in female rats observed by Kirsten et al. 2012 does not necessarily contradict our results.

The presence of repetitive behavior remains one of the core criteria for the diagnosis of autism in the revised DMS-V manual used by many clinicians world-wide. One of the more striking results that we observed in terms of both the magnitude of the effect, and the gender divergence, was on the quantitation of repetitive and stereotyped behaviors. Although other groups have reported elevated repetitive behaviors after MIA in rodents [26], [27], [35], [36], [37], these and other studies most often used males only, or combined data from both sexes [38] for discussion on male bias in immune activation studies).

Our results highlight the male selectivity of this phenomenon in the MIA animal model. Male LPS and Poly IC offspring both showed robust increases in marble burying, whereas female LPS and Poly IC mice showed no significant increase. In addition to increased repetitive behaviors, the same group of male Poly IC-treated mice also showed decreased motor activity as measured by total distance traveled and the number of horizontal movements initiated. The decrease in movements is likely the result of more time spent engaging in stationary, stereotyped behavior.

Schwartzer et al., 2013 examined repetitive behaviors after Poly IC MIA in two strains of mice, the C57/BL6 as used here, and the BTBR mouse, an inbred strain reported to be predisposed to exhibit ASD-like behavior [35]. The Poly IC offspring of both strains showed significantly elevated marble burying compared to saline controls but male and female data were combined. In the grooming test where male and female data were reported separately, increased grooming was observed only in males for both strains (the effect was significant in BTBR mice). Moreover, in the present study, we also examined the offspring from a separate group of mice whose mothers were given a second injection of Poly IC during a second pregnancy after the first Poly IC litter was born. Again, male offspring from the second litter showed a significant increase in marble burying over saline controls, whereas the female mice did not (see Figure S4), further confirming the gender selectivity of stereotyped behavior in the Poly IC MIA model.

The strong sex-bias observed in marble burying and grooming in MIA mice is, in fact, reflected in human ASD patients. Several studies have demonstrated that male autistics have more repetitive and/or restricted interests compared to female autistics [39]–[44]. However, there are also a few published studies that found no gender differences in repetitive behavior in ASD in children and adolescents [45] and in pre-school children with suspected ASD [46]. Overall, we note that the lack of, or lower level of motor stereotypies generally reported in females with ASD, does not preclude the possibility that they may possess other forms of repetitive behavior, such as restricted interests or other forms of ritualistic behavior.

The question as to the nature of the underlying molecular and cellular mechanism(s) linking immune perturbations to ASD remains unanswered. Poly IC is a double-stranded RNA which mimics a viral infection and activates Toll-like Receptor 3 while LPS is a cell wall component of gram negative bacteria that activates Toll-like Receptor 4 [47], [48]. Activation of Toll-like Receptors induces the production of immune proteins including cytokines, and also long non-coding RNAs which regulate the innate immune response [49]. The induction of MIA in rodents has been associated with elevated levels of pro-inflammatory cytokines in the maternal circulation, as well as in the placenta and fetal circulation [38], [50], [51]. It should be noted that another study in mice demonstrated that even in the absence of an immune stimulus, cytokines display dynamic changes in levels over the course of normal development and also that a widespread decrease was detected in several brain cytokines during peak periods of synaptogenesis and plasticity (PND 7–30) [51]. This group also reported low levels of some cytokines after induction of MIA and suggested that aberrantly low cytokine signaling might be a factor causing altered brain connectivity and ASD and/or schizophrenic-like behaviors in the offspring [51]. Finally, within the context of neurodevelopmental disorders, although the mechanisms by which cytokines (and chemokines) [15] might induce deleterious effects is not yet known, one working concept is that there is a disruption after MIA of the normal balance between pro- and anti-inflammatory influences that is critical for normal fetal brain development [23], [52].

In summary, our findings indicate that immune stimulation during fetal development in mice causes differential effects in males vs. females. Intriguingly, our results reveal that MIA induces the selective induction of stereotyped, repetitive behavior in male mouse offspring but not female offspring. This finding is consistent with reports of a higher incidence of repetitive behaviors in male compared to female autistic patients. Although certain brain regions such as the cerebellum and basal ganglia have been associated with repetitive behaviors in both humans with autism and in animal models of autism, and alterations in cytokines could be a contributing factor, future studies should be directed towards further elucidating the molecular mechanisms underlying the gender-selective effects of *in utero* immune activation on repetitive and other autistic-like behaviors.

## Supporting Information

Figure S1 **The effects of maternal immune activation on vertical motor activity of adult offspring.** ANOVA revealed a significant gender effect for first two time intervals (0–20 min: (*F* (1, 107) = 20.77, ###*p*<0.001) and 20–40 minutes (*F* (1, 107) = 6.40, #*p*<0.05). A significant treatment effect was observed in the middle time interval (*F* (2, 107) = 4.91, *p*<0.01), although a post-hoc Bonferroni test did not reveal a significance difference between groups. The treatment x gender interaction effect was not significant for any of the time intervals. Each column represents mean ± S.E.M. The number of mice tested (n) for males (m) and females (f) for each condition is indicated.
(TIF)

Figure S2 **Analysis of thigmotaxis behavior of adult offspring.** Total time in the center zone is shown in panel A, while the total distance travelled in the center zone of the activity box during the first 20 minutes of the exploration period is shown in panel B. No significant treatment effects were seen among the groups. However, a significant gender effect was observed for both tests (#p<0.05; ##p<0.01). Each column represents mean ± S.E.M. The number of mice tested (n) for each condition is indicated.
(TIF)

Figure S3 **The effects of maternal immune activation on social behavior of adult offspring during the three-chamber social preference test.** (A) Diagrammatic depiction of the experimental set-up for the social preference test. (B) No significant preference for the non-familiar zone over the familiar zone was seen in any of the test conditions. Each column represents average ± S.E.M.
(TIF)

Figure S4 **The effects of maternal immune activation on marble burying of adult offspring.** Poly IC 2X offspring were produced from dams that have been injected with 20 mg/kg of Poly IC on E12.5 during two consecutive pregnancies. Two-way ANOVA revealed a significant treatment effect (*F* (1, 84) = 4.87, *p*<0.05), gender effect (*F* (1, 84) = 8.24, *p*<0.01), and treatment x gender interaction effect (*F* (1, 84) = 11.0, *p*<0.01, see Table S1 for statistical details). Further post-hoc test showed that male Poly 2X offspring buried significantly more marbles compared to the respective saline controls, while female Poly 2X offspring showed no significant difference. Each column represents mean ± S.E.M. ##*p*<0.01 (between gender); ***p<0.001 (between treatment).
(TIF)

Table S1 **Summary of statistical analyses. 2-way ANOVA was conducted for Saline, LPS 1X, Poly IC 1X for all behavioural tests with the exception of Figure S4 where 2-way ANOVA analysis was performed for Saline and Poly 2X offspring only.** 3-way ANOVA was performed for motor activity (i.e. Fig. 2 and Figure S1). In cases where the treatment effect was significant but the treatment x gender Interaction effect was not significant, a Bonferroni Post-hoc test was performed for treatment by combining male and female data. In cases where both the treatment effect and treatment x gender interaction effect were significant, a Bonferroni Post-hoc test was performed for treatment for each gender separately. Hor. – horizontal motor activity; Ver. – vertical motor activity; M – Male; F – Female. N/A – not applicable.
(DOCX)
