# Supplementary figures and images for: Gender-Dependent Effects of Maternal Immune Activation on the Behavior of Mouse Offspring
Source: PLoS One. 2014 Aug 11;9(8):e104433. doi: 10.1371/journal.pone.0104433 (PMC4128679; doi:10.1371/journal.pone.0104433)

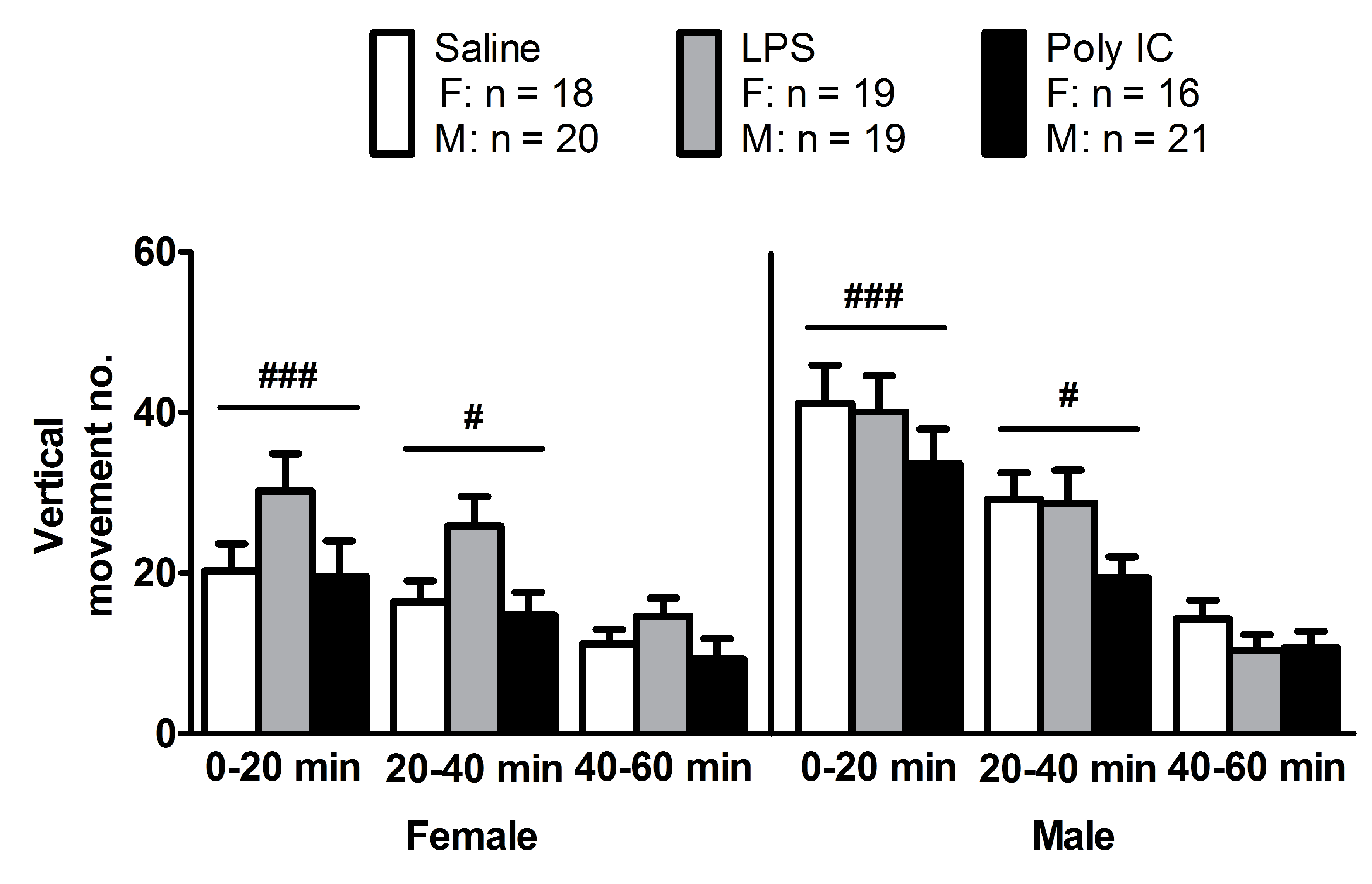

Supplement: Figure S1 — The effects of maternal immune activation on vertical motor activity of adult offspring. ANOVA revealed a significant gender effect for first two time intervals (0–20 min: (F (1, 107) = 20.77, ###p<0.001) and 20–40 minutes (F (1, 107) = 6.40, #p<0.05). A significant treatment effect was observed in the middle time interval (F (2, 107) = 4.91, p<0.01), although a post-hoc Bonferroni test did not reveal a significance difference between groups. The treatment x gender interaction effect was not significant for any of the time intervals. Each column represents mean ± S.E.M. The number of mice tested (n) for males (m) and females (f) for each condition is indicated. (TIF) [file pone.0104433.s001.tif]

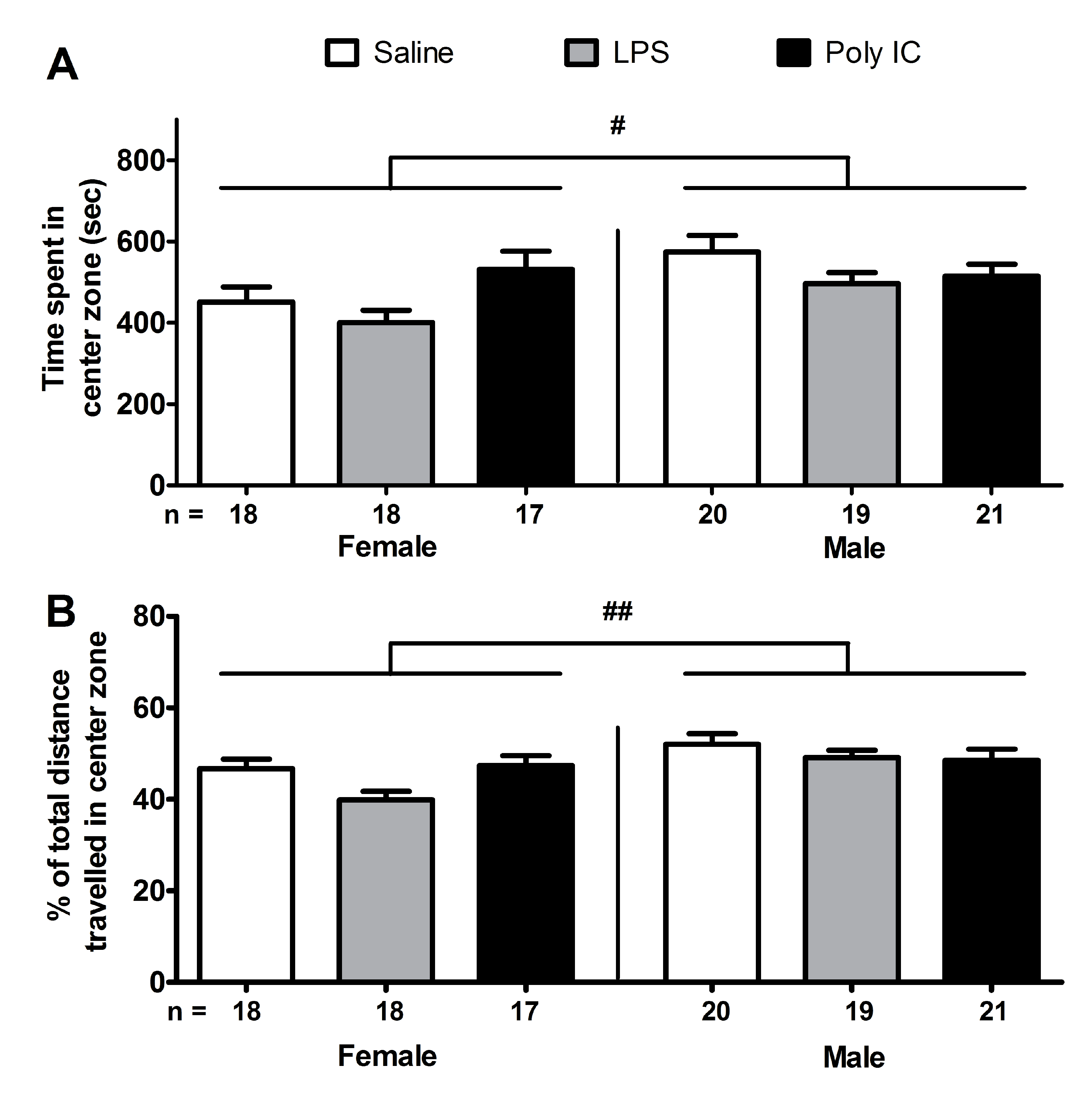

Supplement: Figure S2 — Analysis of thigmotaxis behavior of adult offspring. Total time in the center zone is shown in panel A, while the total distance travelled in the center zone of the activity box during the first 20 minutes of the exploration period is shown in panel B. No significant treatment effects were seen among the groups. However, a significant gender effect was observed for both tests (#p<0.05; ##p<0.01). Each column represents mean ± S.E.M. The number of mice tested (n) for each condition is indicated. (TIF) [file pone.0104433.s002.tif]

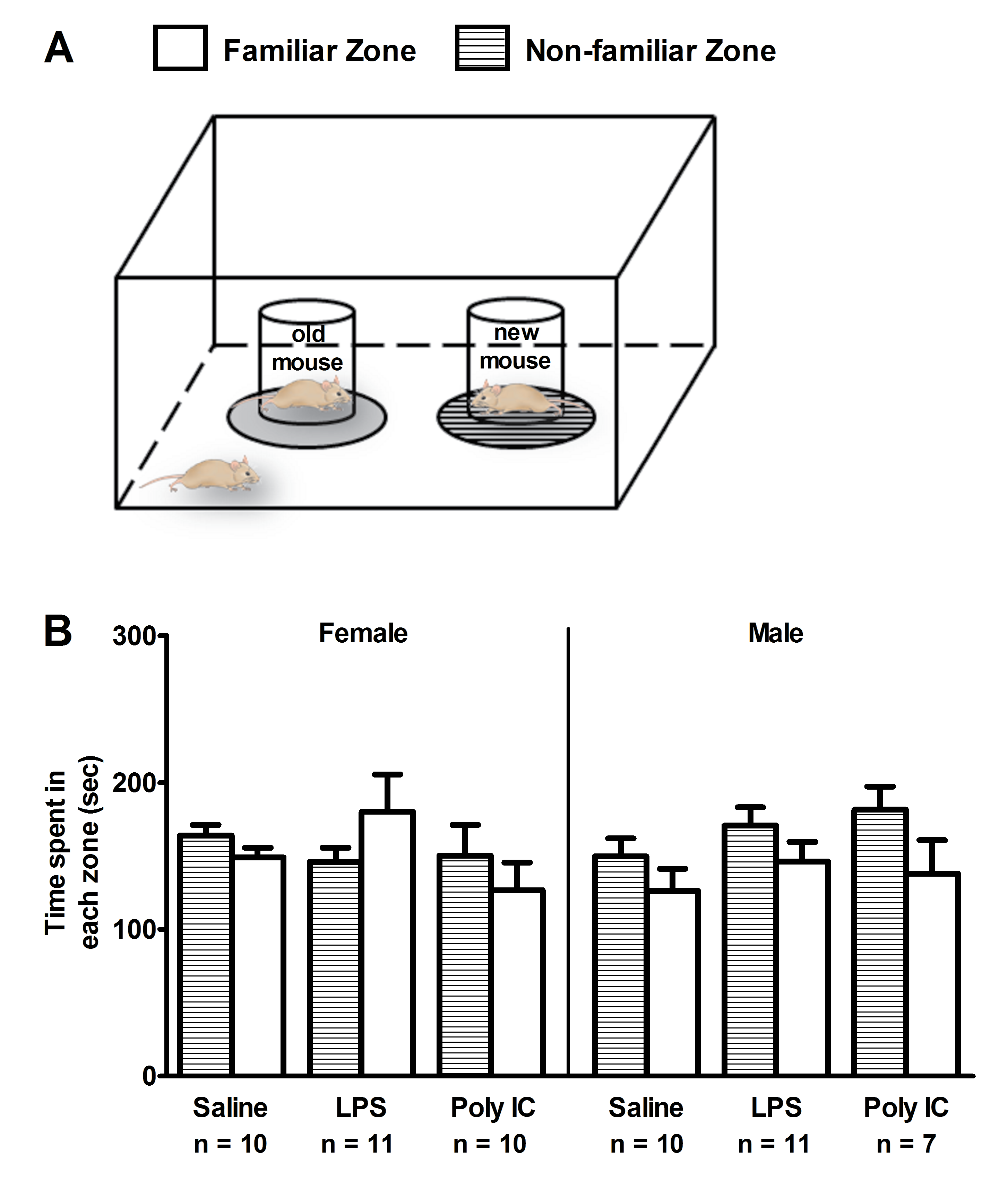

Supplement: Figure S3 — The effects of maternal immune activation on social behavior of adult offspring during the three-chamber social preference test. (A) Diagrammatic depiction of the experimental set-up for the social preference test. (B) No significant preference for the non-familiar zone over the familiar zone was seen in any of the test conditions. Each column represents average ± S.E.M. (TIF) [file pone.0104433.s003.tif]

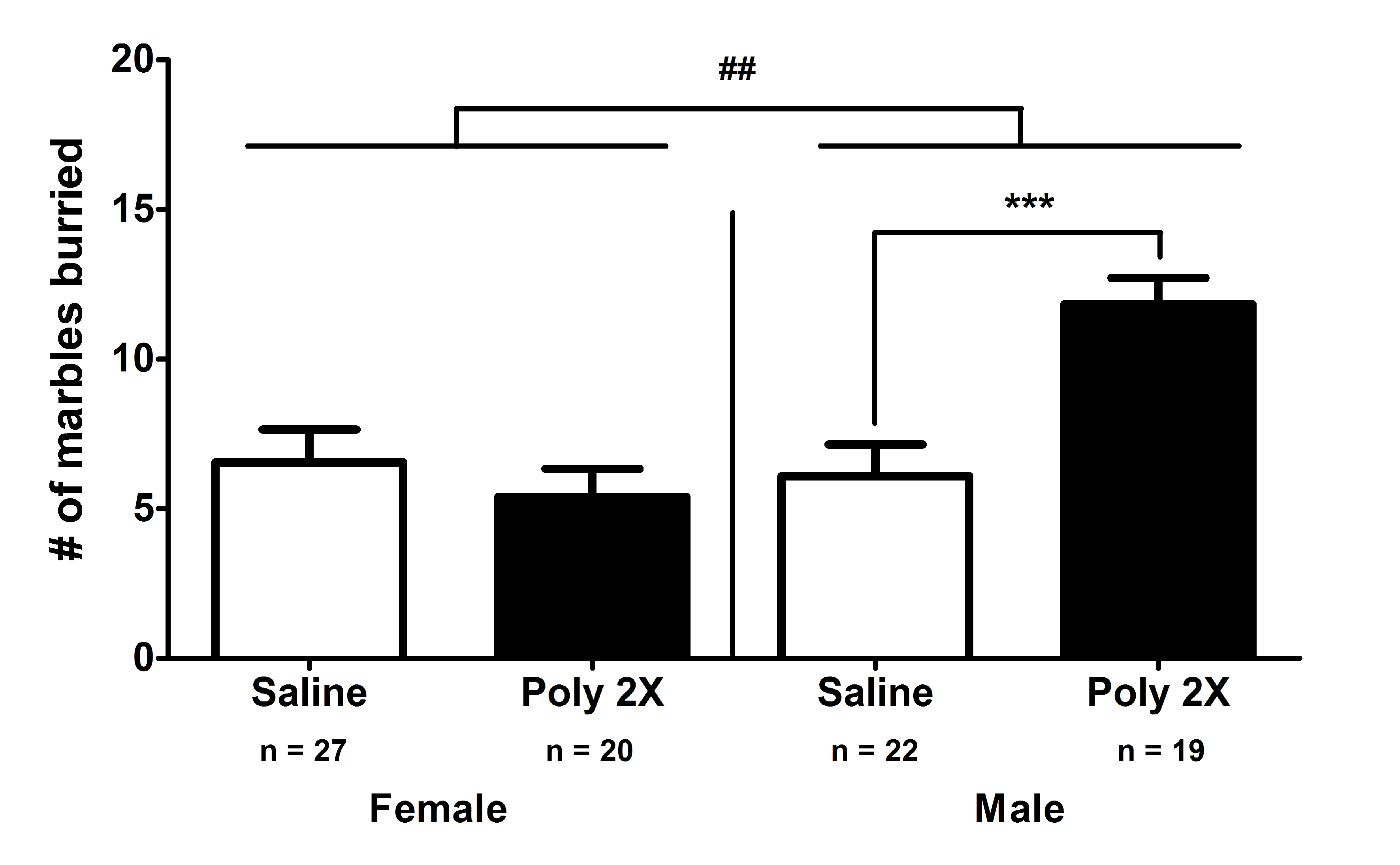

Supplement: Figure S4 — The effects of maternal immune activation on marble burying of adult offspring. Poly IC 2X offspring were produced from dams that have been injected with 20 mg/kg of Poly IC on E12.5 during two consecutive pregnancies. Two-way ANOVA revealed a significant treatment effect (F (1, 84) = 4.87, p<0.05), gender effect (F (1, 84) = 8.24, p<0.01), and treatment x gender interaction effect (F (1, 84) = 11.0, p<0.01, see Table S1 for statistical details). Further post-hoc test showed that male Poly 2X offspring buried significantly more marbles compared to the respective saline controls, while female Poly 2X offspring showed no significant difference. Each column represents mean ± S.E.M. ##p<0.01 (between gender); ***p<0.001 (between treatment). (TIF) [file pone.0104433.s004.tif]
