# Supplementary material for: Gender-Dependent Effects of Maternal Immune Activation on the Behavior of Mouse Offspring
Source: PLoS One. 2014 Aug 11;9(8):e104433. doi: 10.1371/journal.pone.0104433 (PMC4128679; doi:10.1371/journal.pone.0104433)
Supplement: Table S1 — Summary of statistical analyses. 2-way ANOVA was conducted for Saline, LPS 1X, Poly IC 1X for all behavioural tests with the exception of Figure S4 where 2-way ANOVA analysis was performed for Saline and Poly 2X offspring only. 3-way ANOVA was performed for motor activity (i.e. Fig. 2 and Figure S1). In cases where the treatment effect was significant but the treatment x gender Interaction effect was not significant, a Bonferroni Post-hoc test was performed for treatment by combining male and female data. In cases where both the treatment effect and treatment x gender interaction effect were significant, a Bonferroni Post-hoc test was performed for treatment for each gender separately. Hor. – horizontal motor activity; Ver. – vertical motor activity; M – Male; F – Female. N/A – not applicable. (DOCX) [file pone.0104433.s005.docx]

| **Main Figures** | **Treatment**  **Effect** | **Post hoc: treatment Saline vs. Poly IC** | **Post hoc: treatment Saline vs. LPS** | **Post hoc: treatment Poly IC vs. LPS** | **Gender**  **Effect** | **Interaction: Treatment x Gender** | **Interaction: Treatment x Gender x Time (three-way ANOVA)** |
| --- | --- | --- | --- | --- | --- | --- | --- |
| Body Weight | 0.325 | N/A | N/A | N/A | < 0.001 | 0.094 | N/A |
| Fig. 2A Total Distance (0 – 20 min) | 0.191 | N/A | N/A | N/A | 0.162 | 0.225 | 0.931 |
| Fig. 2A Total Distance (20 – 40 min) | < 0.001 | 0.003 | 0.541 | < 0.001 | 0.032 | 0.177 | 0.931 |
| Fig. 2A Total Distance (40 – 60 min) | 0.015 | 0.005 | 0.754 | 0.115 | < 0.001 | 0.395 | 0.931 |
| Fig. 2B Total No. Hor. Movements (0 – 20 min) | 0.417 | N/A | N/A | N/A | 0.968 | 0.209 | 0.841 |
| Fig. 2B Total No. Hor. Movements (20 – 40 min) | < 0.001 | < 0.001 | 0.003 | 1.000 | 0.286 | 0.182 | 0.841 |
| Fig. 2B Total No. Hor. Movements (40 – 60 min) | 0.006 | 0.002 | 0.136 | 0.451 | < 0.001 | 0.694 | 0.841 |
| Fig. 4A Marble Burying Test | 0.005 | M: < 0.001  F: > 0.05 | M: < 0.05  F: > 0.05 | M: > 0.05  F: > 0.05 | 0.199 | 0.020 | N/A |
| Fig. 4B Grooming (Habituation) | 0.030 | 1.000 | 0.056 | 0.373 | 0.002 | 0.446 | N/A |
| Fig. 4B Grooming (Social Preference Test) | 0.037 | 0.592 | 0.585 | 0.028 | 0.428 | 0.797 | N/A |
|  |  |  |  |  |  |  |  |
| **Supplementary Figures** | **Treatment**  **Effect** | **Post hoc: treatment Saline vs. Poly IC** | **Post hoc: treatment Saline vs. LPS** | **Post hoc: treatment Poly IC vs. LPS** | **Gender**  **Effect** | **Interaction: Treatment x Gender** | **Interaction: Treatment x Gender x Time (three-way ANOVA)** |
| Fig. 1 Total No. Ver. Movements (0 – 20 min) | 0.093 | N/A | N/A | N/A | < 0.001 | 0.489 | 0.963 |
| Fig. 1 Total No. Ver. Movements (20 – 40 min) | 0.009 | 0.231 | 0.621 | 0.009 | 0.013 | 0.274 | 0.963 |
| Fig. 1 Total No. Ver. Movements (40 – 60 min) | 0.391 | N/A | N/A | N/A | 0.972 | 0.207 | 0.963 |
| Fig. 3A Center Duration | 0.069 | N/A | N/A | N/A | 0.020 | 0.109 | N/A |
| Fig. 3B % of Total Distance Travelled in Center Zone | 0.069 | N/A | N/A | N/A | 0.003 | 0.156 | N/A |
| Fig. 4 Marble Burying Test (Saline and Poly 2X only) | 0.020 | M: < 0.001  F: > 0.05 | N/A | N/A | 0.002 | 0.006 | N/A |

**Supplementary Table 1.** Summary of statistical analyses. 2-way ANOVA was conducted for Saline, LPS 1X, Poly IC 1X for all behavioural tests with the exception of supplementary fig. 4 where 2-way ANOVA analysis was performed for Saline and Poly 2X offspring only. 3-way ANOVA was performed for motor activity (i.e. Fig. 2 and Supplementary Fig. 1). In case where the treatment effect was significant but the treatment x gender Interaction effect was not significant, Bonferroni Post-hoc test was performed for treatment by combining male and female data. In case where both the treatment effect and treatment x gender interaction effect were significant, Bonferroni Post-hoc test was performed for treatment for each gender separately. Hor. – Horizontal; Ver. – Vertical; M – Male; F – Female. N/A – not applicable.
